# Identifying HIV infection in South African women: How does a fourth generation HIV rapid test perform?

**DOI:** 10.4102/ajlm.v1i1.4

**Published:** 2011-12-15

**Authors:** Kapila Bhowan, Emma Kalk, Sonjiha Khan, Gayle Sherman

**Affiliations:** 1 Paediatric HIV Diagnostic Syndicate, Wits Health Consortium, Johannesburg South Africa; 2 Department of Molecular Medicine and Haematology, Faculty of Health Sciences, University of the Witwatersrand, Johannesburg, South Africa; 3 National Health and Laboratory Service, Johannesburg, South Africa

## Abstract

**Background:**

HIV rapid tests (RT) play an important role in tackling the HIV pandemic in South Africa. Third generation RT that detect HIV antibodies are currently used to diagnose HIV infection at the point of care. Determine Combo (DC) is the first fourth generation RT that detects both p24 antigen (p24Ag) and HIV antibodies (Ab), theoretically reducing the window period and increasing detection rates. Early detection of maternal HIV infection is important to mitigate the high risk of vertical transmission associated with acute maternal infection.

**Objectives:**

We assessed the performance of the DC RT against third generation RT in antenatal and post-partum women.

**Methods:**

Third generation RT Advance Quality and Acon were used in a serial algorithm to diagnose HIV infection in antenatal and post-partum women over six months at a tertiary hospital in Johannesburg, South Africa. This data provided the reference against which the DC RT was compared on plasma and whole blood samples.

**Results:**

The 1019 participants comprised 345 (34%) antenatal and 674 (66%) post-partum women. Ninety women (8.8%) tested HIV-positive of whom 59 (66%) were tested antenatally, and 31 (34%) post-partum yielding prevalence rates of 17.1% and 4.6% respectively. The sensitivity and specificity of the Ab component of DC on plasma antenatally was 100% (93.8% – 100%) and 100% (98.6% – 100%) respectively and post-partum was 100% (88.9% – 100%) and 99.6% (98.8% – 99.9%) respectively. One false positive and not a single true positive p24Ag was detected. Of 505 post-partum women who tested HIV-negative 6–12 months prior to enrolment, 12 (2.4%) seroconverted.

**Conclusion:**

The fourth generation DC offered no advantage over current third generation RT in the diagnosis of HIV infection.

## Introduction

HIV rapid tests (RT) play an important role in addressing the HIV and AIDS pandemic in South Africa. They can be conducted at the point of care because they are easy to perform and require no special instrumentation. The advantage of point of care RT is that the patient can receive their HIV test result at the same clinic visit, which reduces loss to follow-up and fast tracking patients into care.^1^ RT are less costly than laboratory-based assays for antibody (Ab) detection namely HIV Enzyme-linked Immunosorbent Assays (ELISA) and viral detection namely HIV DNA or RNA or p24 Antigen (p24Ag).

**FIGURE 1:**
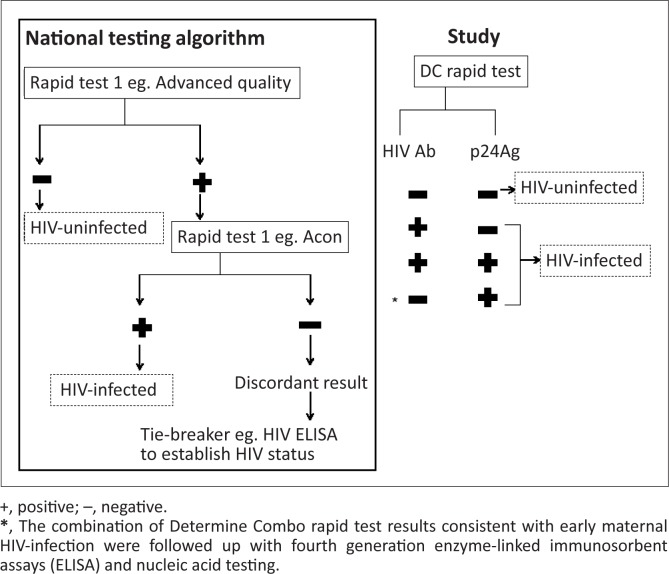
Study methodology: The Determine Combo rapid test (DC RT) was performed at the same time as the routine algorithm.

In South Africa, pregnant women are offered counselling and testing for HIV at their first antenatal clinic visit and at 34 weeks of pregnancy if their initial HIV test was negative.^2^ Women at the Rahima Moosa Mother and Child Hospital (RMMCH) in Johannesburg, South Africa are also offered an HIV test immediately post-partum if their HIV status is unknown or more than six weeks have elapsed since their last negative HIV test. The importance of HIV retesting has been demonstrated by a South African study in which 3.4% of women who tested HIV-negative at their first antenatal visit, subsequently seroconverted during pregnancy or within a year after delivery.^3^ The Advance Quality HIV Rapid Test (In Tec Products, Inc. Xianen, China) and Acon HIV 1/2/0 Tri-line Rapid Test (Acon Laboratories, Inc., San Diego, USA) are currently used to diagnose HIV infection in women in Prevention of Mother-to-Child transmission (PMTCT) programmes in Gauteng province, South Africa. These third generation RT detect HIV Ab that are produced in response to the virus by a serial testing algorithm as recommended by the South African PMTCT guidelines^2^ (Figure 1). Advance Quality is used to screen for HIV Ab and if positive, the Acon test is performed to confirm HIV status. Laboratory-based HIV ELISA and, less commonly, viral detection assays can be used as a tiebreaker to confirm an HIV status if serial RT results are discordant.^2^

The Determine Combo HIV-1/2 Ag/Ab Combo Test (DC) (Inverness Medical, Japan Co.,Ltd) is the first fourth generation RT and can be performed on either plasma or whole blood samples.^4^ It is an enhancement of the third generation tests as it can detect both HIV Ab and p24Ag in a single test.^5^ The p24Ag is a marker of early HIV infection and is detectable in blood during the window period before HIV Ab become detectable.^6^ The DC RT is reported to have the potential to identify HIV infection five days (range 2–20 days) earlier than third generation RT. The reported sensitivity and specificity of the DC Ab component is 100% (95% confidence interval 98% – 100%) and 100% (95% confidence interval 98.2% – 100%) respectively and the sensitivity of the p24Ag component is 86.6% (95% confidence interval 76% – 93.7%).^7^

Detection of early HIV infection during the window period of third generation RT would allow more maternal HIV infections to be detected. Furthermore, during early maternal HIV infection the levels of the virus in the blood are at their peak and the risk of transmission to the infant during birth and breast-feeding is very high.^8^ Detection of early infection would allow more women and their infants at a high risk of vertical transmission to access PMTCT.

The performance of the fourth generation HIV DC RT in diagnosing HIV infection status antenatally and in the early post-partum period in comparison to the third generation HIV RT in routine use at public healthcare facilities in Gauteng, South Africa was assessed. The advantage offered by the DC RT over third generation RT of detecting HIV infection earlier to increase identification of women at a high risk of vertical transmission for PMTCT was investigated.

## Methods

### Study participants

Women attending the antenatal clinic and delivery unit at RMMCH in Johannesburg were invited to test for HIV infection. Counsellors interviewed the participants to establish their HIV status. Women with an unknown HIV status, those who had tested HIV-negative more than 6 weeks previously and those who reported an HIV-positive status but had no documented evidence thereof on their maternal card were invited to participate in the study. Women with a documented HIV-positive status were excluded. Written informed consent was obtained from all participants who agreed to test for HIV infection. Ethics approval (M091119) for the study was granted by the Human Research Ethics Committee at the University of the Witwatersrand, Johannesburg.

### Sample size

The prevalence of HIV infection amongst antenatal women in Gauteng province, South Africa in 2008 was 29.9% (95% confidence interval 28.4% – 31.2%)^9^; however, the prevalence of HIV infection amongst women testing antenatally and shortly after delivery at RMMCH in 2008 was lower at 15% and 4.2% respectively since women with a known, documented HIV-positive status are excluded and proportionately more women fall into this category after delivery than antenatally.^10^ From RMMCH HIV testing records we expected approximately 200 women to present for testing per month with a prevalence that depended on the proportion of women testing before or after delivery. A convenience sample of all women eligible for HIV testing at RMMCH who agreed to participate in the study was chosen to assess the number of additional women the DC RT could identify as being infected over a 6-month period on plasma samples. For assessment of whole blood samples, the Centers for Disease Control and Prevention recommendations to include samples from at least 20 HIV-infected and 80 HIV-uninfected women were followed.^11^

### HIV testing

Five millilitres of whole blood was drawn into an ethylenediaminetetraacetic (EDTA) tube for testing at the study site. Samples were centrifuged to obtain plasma on which the RT were performed. DC RT were performed on whole blood prior to centrifugation. All RT were performed within one hour of blood sampling by a single laboratory technician according to the manufacturer’s instructions. Third generation RT Advance Quality and Acon were used serially on plasma according to the national testing algorithm^2^ to diagnose HIV infection (Figure 1) and were the reference standards against which the DC results were compared.

### Interpretation of rapid test

The DC is a qualitative immunochromatographic test which is read visually. The test strip is divided into an HIV Ab window and an HIV p24Ag window. The presence of a pink line in either or both of the windows is indicative of HIV infection. Each test strip incorporates a procedural positive control and the test is considered valid only if the positive control is detected.

The women received their RT results and post-test counselling within four hours of blood sampling. Patients that were HIV-positive on both third generation RT were referred to antiretroviral treatment clinics. The results of the DC RT were not disclosed to the patient. However, where the third generation and DC HIV Ab RT results were discordant, samples were referred for confirmatory fourth generation ELISA (ARCHITECHT^®^ HIV Ag/Ab Combo assay; Abbott Diagnostics; Wiesbaden, Germany). Patients that had detectable p24 Ag on DC were followed up with three confirmatory tests that is viral detection assay (Vironostika HIV-1 Antigen; bioMerieux; Bosiend, The Netherlands) , viral load testing (NucliSENS EasyQ-EasyMag HIV-1, version 1.2 assay; bioMerieux; Boxtel, The Netherlands) as well as fourth generation ELISA. Disclosure of the patient’s HIV status was delayed for 48 hours.

### Analysis

Likelihood ratios were calculated instead of predictive values because predictive values depend on prevalence and a difference in HIV prevalence was anticipated between women tested in the antenatal and post-partum period. A positive likelihood ratio (sensitivity/(1 – specificity)) > 10 strongly predicts HIV-infection, whereas a negative likelihood ratio ((1 – sensitivity)/specificity) < 0.1 virtually excludes the condition.

## Results

Between March and August 2010, 1019 (92.7%) of the 1099 women eligible for HIV testing at RMMCH were enrolled in the study. Of the 1019 participants, 345 (33.9%) were tested antenatally and 674 (66.1%) post-partum. According to the routine third generation RT diagnostic algorithm, 90 (8.8%) of the 1019 patients tested positive for HIV infection without the need to use a tiebreaker. Of the 90 HIV-infected women, 59 (65.6%) were antenatal and 31 (34.4%) were post-partum. The HIV prevalence amongst the women tested antenatally was 17.1% and those tested post-partum was 4.6%.

Knowledge of the women’s HIV status prior to undergoing HIV testing on the study was documented for those tested in the early post-partum period only (Table 1). Of the 505 women that had reported or tested HIV-negative between 6 and 12 weeks prior to study enrolment, 12 (2.4%) tested positive demonstrating that new HIV infections were occurring in this population. Not all of these women had their negative HIV status documented on their maternal record and the possibility that some reported their status incorrectly cannot be excluded.

**TABLE 1:** HIV status of post-partum women prior to enrolment and after testing with the National testing algorithm.

Reported or documented HIV status	Time since last test (weeks)	*n*	HIV-infected	HIV-uninfected	Percentage positive
Negative	6–12	505	12	492	2.4
	> 12	107	3	104	2.8
Unknown HIV status	-	58	14	43	24.1
Positive: reported but not documented	-	4	2	2	50.0
**Total**	**-**	**674**	**31**	**641**	**4.6**

*n*, sample size.

Women of unknown HIV status had a high prevalence of HIV infection. Half of the women who reported a positive HIV status but had no documentation to substantiate a positive HIV test, tested HIV-negative.

The DC RT was performed on plasma samples of the 1019 women and on whole blood samples on a subset of 380 women. All 1399 tests performed demonstrated positive control strips therefore no DC RT was invalid. Sensitivity, specificity and likelihood ratios for the DC RT were calculated separately for women who were tested antenatally and post-partum (Table 2). The sensitivity of the DC RT Ab component was 100% in all groups of women tested irrespective of the sample type. The specificity of the DC RT Ab component in women tested post-partum was less than those tested antenatally.

**TABLE 2:** Performance of Determine Combo rapid test HIV antibody (DC RT Ab) component in plasma and whole blood samples in women tested antenatally and post-partum.

Time test was conducted	Sample type	*n*	Prevalence	Determine Combo HIV Ab+/HIV+	Sensitivity	95% confidence interval	LR+	Determine Combo HIV Ab-/HIV-	Specificity	95% confidence interval	LR-
Antenatal	Plasma	345	17.1	59/59	100	93.8–100.0	569.2	286/286	100.0	98.6–100.0	0
	Whole blood	151	-	59/59	100	93.8–100.0	184.5	92/92	100.0	95.9–100.0	0
Post-partum	Plasma	674	4.6	33/31^†^	100	88.9–100.0	321.5	641/643	99.7	98.9–99.9	0
	Whole blood	229	-	32/30^†^	100	88.6–100.0	99.5	197/199	99.0	96.4–99.7	0

*n*, sample size.

† False positive results obtained in 2 women on both sample types. One additional women in this group tested HIV p24Ag false positive on DC.

HIV+ and HIV- are HIV-infected and HIV-uninfected women respectively as defined by the third generation rapid test (RT) algorithm.

LR+ and LR- are the positive and negative likelihood ratios.

The high positive likelihood ratios (LR+) and low negative likelihood ratios (LR-) demonstrate that the Determine Combo rapid test was a strong predictor of HIV-infection in HIV positive women and excluded HIV infection in HIV-uninfected women respectively, regardless of sample type and timing of testing.

The DC RT results concurred with the third generation RT results in all patients except for three post-partum women on whom both plasma and whole blood samples were tested. In two patients the Ab component of the DC RT was positive on both their plasma and whole blood samples but negative on both third generation RT. Laboratory based fourth generation ELISA tests on both cases were negative confirming two false positive DC HIV Ab results.

The DC p24Ag was reactive on plasma and the whole blood of only one patient of all 1019 women tested. This patient was suspected of having an early infection since both third generation RT and the DC Ab component tested negative. However the laboratory based fourth generation HIV ELISA was negative and the viral load was undetectable indicating a false positive DC p24Ag result. No p24Ag bands on DC were obtained on any of the 90 HIV-infected women nor was a single case of early HIV infection detected.

## Discussion

Considering the high enrolment rate, the sample of women tested in this study is likely to be representative of women who are tested for HIV-infection at RMMCH over half a year. Furthermore, the HIV prevalence of 17.1% and 4.6% in women testing antenatally and immediately post-partum respectively is similar to a previous description at RMMCH.^10^ An HIV prevalence of 24.1% in women of unknown HIV status is also comparable to the 28% prevalence previously described in 2008. However, the seroconversion rate in post-partum women of 2.4% is less than the previously described rate of 4.5%.^10^ The implication that new maternal infections are occurring in this population remains. In practice, a positive p24Ag DC test would require confirmation of early seroconversion by fourth generation ELISA or nucleic acid testing which may delay initiation of PMTCT. In contrast to a study that reported 16% invalid DC RT tests due to failure to detect the control,^12^ all DC RT in this study were valid possibly because we used fresh, not stored samples.

Sensitivity and specificity of the DC RT was comparable to plasma and whole blood in contrast to a previous report that demonstrated lower sensitivity of the DC RT in whole blood compared to serum samples.^12^ The sensitivity of the fourth generation DC RT in detecting HIV Ab was comparable to that of the reference third generation RT in antenatal and post-partum women in plasma and whole blood samples. The specificity of the DC RT in detecting HIV Ab was slightly reduced in whole blood and plasma in post-partum women owing to the false positive results in two women, but was still within the World Health Organization recommended range of more than 98%.^13^

The fourth generation DC RT did not detect a single true positive p24Ag, even in the 90 HIV-infected women. The sensitivity in detecting p24Ag in HIV-infected women was 0% as compared with the claim of 86.6% obtained on HIV-infected samples.^7^ The reason for this may be that p24Ag forms immune complexes with HIV Ab and thus no free p24Ag is present for detection by the DC.^6^ The DC RT therefore did not identify any new cases of maternal HIV infection over the 6 month study period. Possible reasons for this include that the sensitivity of the DC p24 Ag component is poor or that no women with acute HIV infection were enrolled. The former concurs with previous reports that the DC RT p24 Ag component lacks sensitivity particularly where levels of p24 Ag are below 400 pg/mL.^12,14^ Additionally, the p24Ag component of the DC RT has reduced sensitivity in comparison to other fourth generation viral detection assays,^7^ some of which are able to detect p24Ag at levels of 4 pg/mL – 5 pg/mL.^15^ The initial laboratory based p24Ag assays also demonstrated poor sensitivity that was subsequently improved by denaturation of the immune complex and signal amplification to enhance p24Ag detection.^16^

A limitation of this study is that the incidence of HIV infection in women undergoing HIV testing at RMMCH is unknown therefore, although new maternal infections were demonstrated, it is possible that no women with acute HIV infection were enrolled during the short window period before HIV Ab and subsequent immune complex formation. Nevertheless, the DC RT did not identify any new infections over those identified by the 3rd generation RT assays over six months in the RMMCH PMTCT programme.

## Conclusion

The DC RT failed to demonstrate any advantage over third generation RT currently in use in our setting in either determining HIV infection status or in identifying recently infected women. Improved sensitivity of p24 Ag detection is required before fourth generation RT will offer an advantage over their third generation counterparts in the field.
